# *Mycobacterium tuberculosis* exploits the PPM1A signaling pathway to block host macrophage apoptosis

**DOI:** 10.1038/srep42101

**Published:** 2017-02-08

**Authors:** Kaitlyn Schaaf, Samuel R. Smith, Alexandra Duverger, Frederic Wagner, Frank Wolschendorf, Andrew O. Westfall, Olaf Kutsch, Jim Sun

**Affiliations:** 1 Department of Medicine, University of Alabama at Birmingham, Birmingham, Alabama, USA

## Abstract

The ability to suppress host macrophage apoptosis is essential for *M. tuberculosis* (*Mtb*) to replicate intracellularly while protecting it from antibiotic treatment. We recently described that *Mtb* infection upregulated expression of the host phosphatase PPM1A, which impairs the antibacterial response of macrophages. Here we establish PPM1A as a checkpoint target used by *Mtb* to suppress macrophage apoptosis. Overproduction of PPM1A suppressed apoptosis of *Mtb-*infected macrophages by a mechanism that involves inactivation of the c-Jun N-terminal kinase (JNK). Targeted depletion of PPM1A by shRNA or inhibition of PPM1A activity by sanguinarine restored JNK activation, resulting in increased apoptosis of *Mtb*-infected macrophages. We also demonstrate that activation of JNK by subtoxic concentrations of anisomycin induced selective apoptotic killing of *Mtb*-infected human macrophages, which was completely blocked in the presence of a specific JNK inhibitor. Finally, selective killing of *Mtb*-infected macrophages and subsequent bacterial release enabled rifampicin to effectively kill *Mtb* at concentrations that were insufficient to act against intracellular *Mtb*, providing proof of principle for the efficacy of a “release and kill” strategy. Taken together, these findings suggest that drug-induced selective apoptosis of *Mtb*-infected macrophages is achievable.

Apoptosis, the process of programmed cell death, is fundamental for the proper maintenance of many biological processes including embryonic development, cell differentiation, and immune system development and regulation^1^. In the context of infectious diseases, both pathogen-induced apoptosis of host cells and the ability of pathogens to suppress host cell apoptosis play an important role in determining disease progression.

This is the case in *Mycobacterium tuberculosis* (*Mtb*) infections, where the ability of the pathogen to control the timing and mode of host cell death plays a pivotal role for *Mtb* persistence and replication^2^,^3^. It is well established that *Mtb* infection suppresses host cell apoptosis to replicate inside the phagosome of infected macrophages^3^,^4^. On the host cell side, apoptosis of infected macrophages has been shown to facilitate intracellular bacterial killing, priming of cell mediated immunity, and limits unnecessary tissue inflammation^2^,^3^,^5^,^6^,^7^. Apoptotic bodies containing *Mtb* are scavenged by activated macrophages and taken up by dendritic cells to facilitate the priming of antigen specific T cells to stimulate adaptive immunity^8^,^9^,^10^. In contrast, the loss of membrane integrity that defines necrosis is used by *Mtb* to exit macrophages, to evade the host immune defenses, and to disseminate^3^,^11^. The ability to prevent macrophage apoptosis is thus essential for the ability of *Mtb* to replicate and persist in its human host. In extension, the ability to modulate cell death could have immense therapeutic potential for the treatment of *Mtb* infections^12^,^13^. A clear mechanistic understanding of the host signaling pathways exploited by *Mtb* to inhibit macrophage apoptosis would allow for the development of targeted therapeutics aimed to restore the ability of macrophages to undergo apoptosis, leading to selective elimination of *Mtb*-infected macrophages.

The majority of previous studies investigating the regulation of host cell death in response to *Mtb* infection have focused on mycobacterial proteins, which resulted in the identification of multiple virulence factors (nuoG^14^, SecA2^15^, pknE^16^, ndkA^17^, cpnT^11^,^18^) that interfere with macrophage cell death^19^. However, research from the host cell perspective is lacking despite the knowledge that *Mtb* infection can regulate apoptosis through both extrinsic and intrinsic pathways by release of cytokines or modulation of the mitochondrial membrane permeability^20^. Evidence has been accumulating that host eicosanoids play an important role in the regulation of *Mtb*-mediated macrophage cell death as it was found that *Mtb* infection induces lipoxin A4 expression, which downregulates the pro-apoptotic and necrosis-protecting prostaglandin E2^9^,^10^. While these pathways are known to affect cell fate, the upstream signals and molecular regulators that control these processes in the context of *Mtb* infection remain largely unknown.

We here demonstrate that the Protein Phosphatase, Mg^2+^/Mn^2+^-dependent 1A (PPM1A), which we recently identified as a key regulator of the innate antibacterial and antiviral response in macrophages^21^, is targeted by *Mtb* to prevent host macrophage apoptosis.

Host serine/threonine phosphatases are known to play important roles for regulation of cellular apoptosis^22^,^23^, and this has been extensively described as potential drug targets in the cancer field^24^,^25^. However, phosphatases have not received much attention in the context of infectious diseases or more specifically pathogen-mediated host cell apoptosis. Kinome analysis provided us with a basic understanding of the protein-protein interaction network governed by PPM1A and allowed us to identify pharmacologically addressable targets to bring proof of principle that therapeutic restoration of the ability of macrophages to undergo apoptosis in response to *Mtb* infection can be achieved. Beyond this, we demonstrate that selective killing of *Mtb*-infected macrophages increased the efficacy of the first-line anti-tuberculosis drug rifampicin, which has been reported to be less effective against intracellular *Mtb*^26^,^27^ due to inefficient penetration into cells^28^,^29^. As such, a “release and kill” strategy to deprive the replicative niche of *Mtb* by inducing *Mtb*-infected macrophage cell death would be a means to more efficiently expose the bacteria to already existing *Mtb* drugs, thereby shortening the currently long treatment times.

## Results

### PPM1A inhibits the macrophage apoptosis pathways

Apoptosis regulation is a critical component of the antibacterial response that has clear implications on pathogen clearance, stimulation of cell mediated immunity, and ultimately disease progression^2^,^3^,^5^,^6^. Kinome analysis of persistently *Mtb*-infected THP-1 macrophages that had guided our previous research on the effect of PPM1A expression on the innate antibacterial response of macrophages^21^, also suggested a possible link of *Mtb*-induced upregulation of PPM1A with the downregulation of a number of proteins with functions in the apoptosis pathways^21^. Indeed, there is precedence that phosphatases play important roles in the regulation of cell death^22^,^23^,^30^.

**Figure 1:**
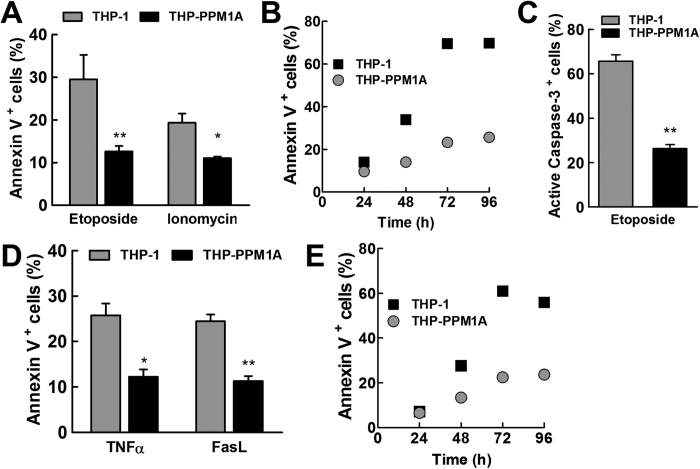
PPM1A overexpression inhibits intrinsic and extrinsic apoptotic pathways. (**A**) THP-1 or THP-PPM1A cells were stimulated with 300 nM etoposide or 10 μM ionomycin for 48 h and 24 h, respectively. Then, cells were stained with Annexin V and analyzed by flow cytometry to quantify the amount of apoptotic cells. (**B**) THP-1 or THP-PPM1A cells were stimulated with 300 nM etoposide for a time course of 24–96 h. Samples were stained every 24 h with Annexin V and analyzed by flow cytometry. (**C**) The amount of apoptotic cells was determined following treatment with 1 μM etoposide for 24 h by the FLICA caspase-3 assay and flow cytometry. (**D**) THP-1 or THP-PPM1A cells were stimulated with 100 ng/ml TNFα or 1 μg/ml FasL for 48 h, and thereafter stained with Annexin V and analyzed by flow cytometry. (**E**) Cells were stimulated with 200 ng/ml TNFα and analyzed as in (**B**). Data in (**A,C,D**) represent the means ± S.D. of three independent experiments. *p < 0.05; **p < 0.01 relative to THP-1 control cells.

Cellular apoptosis can be induced by intrinsic (cell stress events leading to collapse of mitochondria membrane potential) and extrinsic (death ligand mediated) signals, leading to the eventual cleavage and activation of caspase 3, the executioner caspase^31^. As THP-1 cells are the most commonly used human monocyte/macrophage model, including frequent use for *Mtb* infection experiments^32^,^33^, we addressed the question whether upregulation of PPM1A, as observed in *Mtb* infection^21^, could prevent macrophage apoptosis through either of these apoptosis pathways using genetically manipulated THP-1 cells. To induce the intrinsic apoptotic death pathway, we used etoposide, a topoisomerase II inhibitor^34^, and ionomycin, a calcium ionophore^35^. A single addition of etoposide at 0.3 μM induced 30% apoptosis in THP-1 cells after 48 h, but only 13% in THP-1 cells overexpressing PPM1A (THP-PPM1A), as measured by Annexin V assays (Fig. 1A). Etoposide-induced apoptosis levels increased as a function of time. After 96 h, etoposide induced apoptosis in >60% of the THP-1 cells, but THP-PPM1A cells remained protected (<25% apoptotic cells) (Fig. 1B). A single ionomycin treatment at 10 μM for 24 h induced 20% apoptosis in THP-1 cells, but only 11% in THP-PPM1A cells (Fig. 1A). As Annexin V staining is mostly suitable for the detection of early apoptotic events, we also stained for active caspase-3 as an alternative indicator for apoptosis using the Fluorochrome Inhibitor of Caspases (FLICA) method^36^. When apoptosis levels were measured by FLICA caspase-3 assays, cells stimulated with 1 μM etoposide for 24 h showed that 66% of THP-1 cells stained positive for active caspase-3, while only 26% of THP-PPM1A cells stained positive for active caspase-3 (Fig. 1C), confirming the previous results.

We found PPM1A to also be involved in the control of the extrinsic apoptosis pathway. TNFα and Fas Ligand (FasL) are two death ligands that induce apoptosis through the extrinsic pathway. A single addition of TNFα (100 ng/ml) or FasL (1 μg/ml) for 48 h induced apoptosis in ~25% of THP-1 cells, but in less than 15% of THP-PPM1A cells (Fig. 1D). Again, in TNFα-treated THP-1 cells, we observed a time-dependent increase in the frequency of apoptotic events to >60%, whereas THP-PPM1A cells were mostly apoptosis-resistant with the percentage of apoptotic cells increasing to only ~20% (Fig. 1E). Thus, PPM1A appears to play an important role in the control of macrophage apoptosis as its expression directly inhibited both intrinsic and extrinsic apoptotic pathways.

### PPM1A expression inhibits apoptosis of *Mtb*-infected macrophages

**Figure 2:**
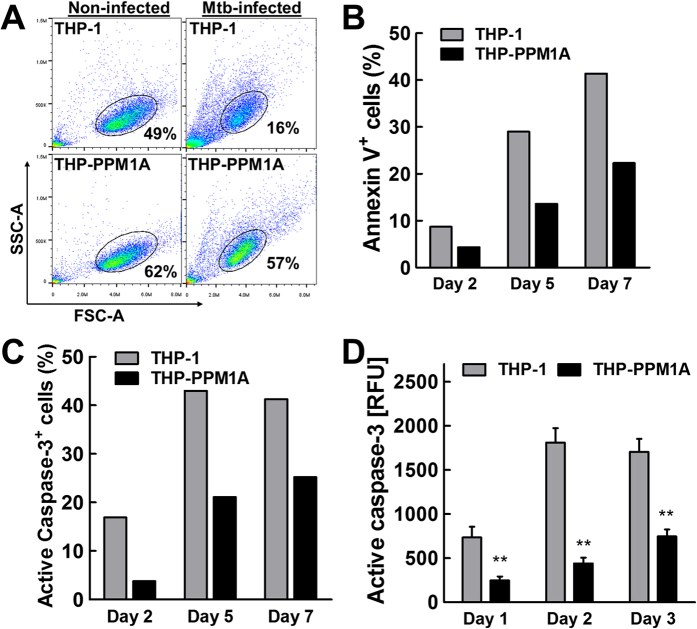
PPM1A inhibits *Mtb*-infection induced apoptosis. (**A**) THP-1 or PPM1A overexpressing THP-1 (THP-PPM1A) cells were infected with *Mtb* at an MOI of 20. 48 h post-infection, cells were harvested for flow cytometry analysis using forward (FSC-A) and side scatter (SSC-A) parameters. Elliptical gate shows percentage of viable cells as determined by scattering profile. (**B,C**) THP-1 or THP-PPM1A cells were infected with *Mtb* at an MOI of 20 to induce apoptosis. At 2, 5 and 7 days post-infection, cells were harvested and stained with (**B**) Annexin V or (**C**) FLICA capsase-3 and analyzed by flow cytometry to measure the amount of apoptotic cells. Results in (**B,C**) are representative of three independent experiments. (**D**) THP-1 and THP-PPM1A cells were infected with *Mtb* as in (**B,C**) and the Apo-ONE assay was used to quantify relative levels of apoptosis. Relative fluorescence units (RFU) correlate to amount of caspase-3/7 activity in each sample, and represent the means ± S.D. of three individual wells. **p < 0.01 relative to THP-1 control cells.

We next examined whether PPM1A plays a role during *Mtb* infection to control macrophage apoptosis. As it has been previously shown that a higher multiplicity of infection (MOI) induces macrophage apoptosis^37^, we infected THP-PPM1A cells with *Mtb* at an MOI of 20. In this scenario, PPM1A overexpressing cells retained higher cell viability relative to parental THP-1 cells (57% vs. 16%), as assessed by flow cytometry on day 2 post infection (Fig. 2A). These results show that increased PPM1A expression levels have a clear impact on the survival of THP-1 cells during *Mtb* infection.

To detail this finding, we next addressed whether the observed *Mtb*-induced cell death was indeed caused by apoptosis. THP-1 or THP-PPM1A cells were infected with *Mtb* at an MOI of 20 and cells were stained for Annexin V at 2, 5, and 7 days post-infection. Over this period of time, apoptosis levels, as determined by Annexin V stains, increased continuously until 40% of THP-1 cells exhibited an apoptotic phenotype on day 7 post-infection (Fig. 2B). In contrast, only ~ 25% of THP-PPM1A cells underwent apoptosis during the same period (Fig. 2B). FLICA caspase-3 staining showed a similar reduction in apoptosis of *Mtb*-infected THP-PPM1A cells in comparison to parental THP-1 cells at the same time points post infection (Fig. 2C). These data show that increased PPM1A levels suppress apoptosis of *Mtb*-infected macrophages. While PPM1A has a major impact on macrophage apoptosis, it does not completely abrogate the apoptotic response, which would be consistent with reports for the involvement of other host-derived or bacterial factors that act outside of the PPM1A signaling axis^14^,^38^.

Measuring apoptosis over time by single cell staining techniques (FLICA, Annexin V) does not provide information on accumulative effects, as cells that underwent apoptosis at earlier time points disintegrate and are not accounted for at later time points. Since *Mtb* infection can also induce macrophage necrosis^11^,^18^, which could result in false positive Annexin V signals or the loss of active caspase-3 signal, both arising from plasma membrane destruction and eventual cellular breakdown, we wanted to confirm our findings with another method for the detection of apoptosis. We thus utilized the Apo-ONE assay^39^, which measures caspase-3/7 activity in a homogenous format following lysis of cells without the need to remove culture media that may already contain released caspase-3/7. The results using this method confirmed that PPM1A overexpression indeed inhibited *Mtb*-induced THP-1 cell apoptosis, as indicated by 4-fold reduction in active caspase-3/7 in the samples from THP-PPM1A cells on day 2 post-infection (Fig. 2D). These data collectively show that elevated PPM1A levels, as observed during persistent *Mtb* infections^21^, inhibit macrophage apoptosis.

### PPM1A controls macrophage apoptosis through JNK activation

Our results clearly demonstrate that PPM1A controls both the extrinsic and the intrinsic apoptotic pathways of macrophages. However, PPM1A has not been previously linked to macrophage apoptosis, which suggests that PPM1A must control downstream effectors that directly mediate the apoptotic response. Given the diversity of proteins that have been reported to be involved in regulation of apoptosis, we used kinome analysis to identify a possible link between PPM1A and proteins known to be involved in the control of apoptosis pathways. The utilized Kinexus antibody array provide information on changes in the expression or phosphorylation state of 309 protein kinases, 38 protein phosphatases, 37 stress response proteins, 24 transcription factors, and 109 proteins involved in other signaling pathways.

Kinome analysis of THP-PPM1A cells in comparison to THP-1 cells revealed a total of 131 statistically relevant signals, which indicate up- or down-regulated protein expression or phosphorylation events. Of these, 78 (59%; p = 1.06e-35) proteins were associated with the regulation of apoptotic processes as indicated by Gene Ontology (GO) process analysis (Table 1). Using the proteins identified from this analysis as seed nodes, we generated a protein-protein interaction network (PIN) using Metacore and identified several nodes that could potentially function as downstream signaling effectors of PPM1A (Supplemental Fig. 1). This analysis identified several highly linked nodes in the JNK-AP1 pathway (c-Jun N-terminal kinase) (Supplemental Fig. 1). As JNK had been previously reported to be a substrate of PPM1A activity^40^ and is known to play a central role in the control of the cellular extrinsic and intrinsic apoptosis pathways^41^, it made for a particularly interesting candidate that could act as a mediator of PPM1A signaling into the apoptotic pathway.

**Figure 3:**
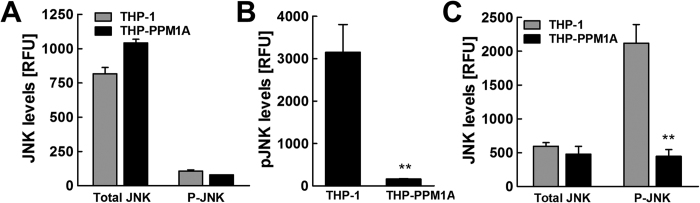
PPM1A control of apoptosis is mediated through inactivation of JNK. (**A**) Resting THP-1 or THP-PPM1A cells were lysed and the amount of total and activated JNK (phosphorylated; T183/Y185) were measured using a 2-plex Milliplex assay kit. RFU indicates relative levels of total or phosphorylated JNK in these cells at baseline. (**B**) THP-1 or THP-PPM1A cells were stimulated with 1 μM anisomycin for 2 h to induce activation of JNK. Thereafter, cells were lysed and levels of phosphorylated JNK were measured by the Milliplex assay. (**C**) THP-1 and THP-PPM1A cells were infected with *Mtb* at an MOI of 20 for 48 h. Then, cells were lysed and the amount of total and activated JNK were measured using the Milliplex assay. Data in this figure represent the means ± S.D. of three independent experiments. **p < 0.01 relative to THP-1 control cells.

Using a multiplex bead assay with specific antibodies to total or phosphorylated JNK (Thr183/Tyr185), our data show similar baseline protein levels of JNK and no significant activation (phosphorylation) in either resting THP-1 or THP-PPM1A cells (Fig. 3A). Treatment of THP-1 cells with anisomycin, a potent JNK activator^42^, induced activation (phosphorylation) of JNK, whereas JNK activation was completely abrogated in THP-PPM1A cells (Fig. 3B). Importantly, following *Mtb* infection of THP-1 and THP-PPM1A cells, while total JNK protein levels remained similar, *Mtb* infection induced JNK activation (phosphorylation) in THP-1 cells, but not in THP-PPM1A cells (Fig. 3C). As PPM1A has also been reported to inactivate p38 MAPK^40^, we examined total and phosphorylated p38 levels in resting and *Mtb*-infected THP-1 macrophages. We found that p38 levels were unchanged at baseline and after *Mtb*-infection in THP-1 and THP-PPM1A macrophages (Supplemental Fig. 2), suggesting that p38 does not play a role in mediating cell death in our system. These data are consistent with the known role of JNK phosphorylation in pro-apoptotic signaling^41^, and thus link PPM1A to apoptosis control at the level of JNK phosphorylation.

### Depletion of PPM1A promotes selective apoptosis of *Mtb*-infected macrophages

**Figure 4:**
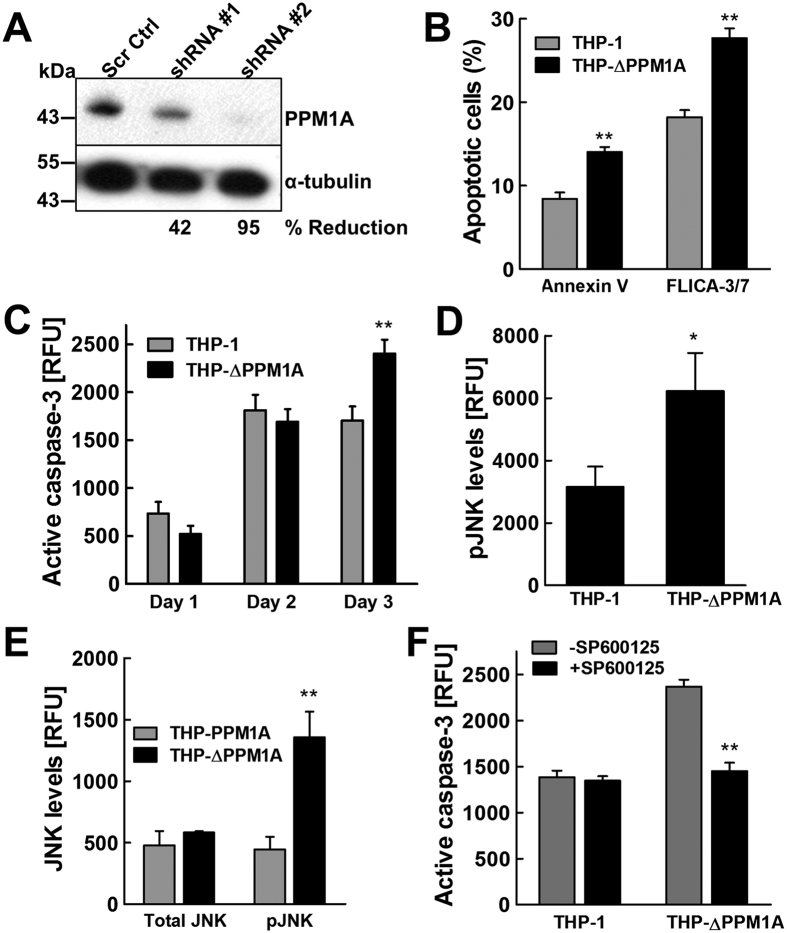
Depletion of PPM1A restores the ability of macrophages to undergo *Mtb*-induced apoptosis. (**A**) THP-1 cells were transduced with lentiviral vectors expressing shRNA targeting PPM1A, or scrambled shRNA (Scr Ctrl). Cell lysates were prepared and PPM1A protein levels were analyzed by Western blotting. Densitometry analysis was performed by ImageJ to quantify PPM1A band intensities as normalized to α-tubulin, and % reduction of PPM1A levels are expressed relative to the control THP-1 cells. (**B**) THP-1 and THP-ΔPPM1A cells were infected with *Mtb* at an MOI of 5. At 3 days post-infection, cells were stained with Annexin V and FLICA capsase-3 and analyzed by flow cytometry to measure the amount of apoptotic cells. (**C**) THP-1 and THP-ΔPPM1A cells were infected with *Mtb* (MOI 5) and the Apo-ONE assay was used to quantify relative levels of apoptosis. Relative fluorescence units (RFU) correlate to amount of caspase-3/7 activity in each sample, and represent the means ± S.D. of three individual wells. (**D**) THP-1 or THP-ΔPPM1A cells were stimulated with 1 μM anisomycin for 2 h to induce activation of JNK. Thereafter, cells were lysed and levels of phosphorylated JNK were measured by the Milliplex assay. (**E**) THP-PPM1A and THP-ΔPPM1A cells were infected with *Mtb* at an MOI of 20 for 48 h. Then, cells were lysed and the amount of total and activated JNK were measured using the Milliplex assay. (**F**) THP-1 and THP-ΔPPM1A cells were infected with *Mtb* (MOI of 5) and at the same time mock treated or treated with 20 μM SP600125 to block JNK activation. At 3 days post infection, relative levels of apoptosis were quantified with the Apo-ONE assay, which measures the amount of caspase-3/7 activity. Data in this figure represent the means ± S.D. of three independent experiments. *p < 0.05; **p < 0.01 relative to THP-1, THP-PPM1A control cells, or non-drug treated cells.

Since *Mtb* infection induces elevated levels of PPM1A expression, which in turn inhibits macrophage apoptosis in response to pathogen invasion, inhibition of PPM1A signaling may be an attractive therapeutic strategy to selectively kill *Mtb*-infected macrophages by inducing infected macrophages to undergo apoptosis. To address this possibility, we generated PPM1A knockdown cells. Transduction of THP-1 cells with a PPM1A-specific shRNA vector produced a cell population (THP-ΔPPM1A cells) in which PPM1A protein levels were reduced to ~10% of the protein levels in parental THP-1 cells (Fig. 4A).

If PPM1A inhibitors, as hypothesized, would trigger selective apoptosis of *Mtb*-infected macrophages, we would expect *Mtb* infection to trigger increased apoptosis in THP-ΔPPM1A cells compared to parental THP-1 cells. To observe the effect of PPM1A depletion on apoptosis of *Mtb*-infected macrophages, we used an MOI of 5, a physiologically representative scenario where *Mtb* infection would naturally suppress host macrophage apoptosis. THP-1 and THP-ΔPPM1A cells were infected with *Mtb* and relative levels of apoptosis were measured by Annexin V, FLICA caspase-3 staining, and Apo-ONE assay. In line with our predictions, using three different methods, we indeed observed a 67% (Annexin V), 52% (FLICA) or 40% (Apo-ONE) increase in *Mtb*-infection induced apoptosis in THP-ΔPPM1A cells, relative to the parental THP-1 cells (Fig. 4B,C).

Our data would suggest that the underlying cause for this increased ability of *Mtb*-infected THP-ΔPPM1A cells to undergo apoptosis would be the restoration of the JNK pathway to respond to the incoming infection. Consistent with our data that PPM1A overexpression would abrogate anisomycin induced JNK activation (Fig. 3B), THP-ΔPPM1A cells stimulated with 1 μM anisomycin showed a 2-fold increase in JNK phosphorylation compared to THP-1 cells (Fig. 4D), adding further evidence that PPM1A expression levels effectively control JNK activation in macrophages. Importantly, different from THP-PPM1A cells, where *Mtb* infection did not induce JNK activation (Fig. 3C), *Mtb* infection induced potent JNK activation in THP-ΔPPM1A cells (Fig. 4E).

To conclusively demonstrate that JNK functions in the PPM1A signaling pathway in the context of *Mtb* infection, we examined whether increased apoptosis associated with the absence of PPM1A (Fig. 4B,C) would actually depend on JNK activation. To this end, we treated *Mtb*-infected THP-ΔPPM1A macrophages with the specific JNK inhibitor SP600125, which has 300-fold selectivity over p38 and no effect on p38 phosphorylation at the concentration used in our experiement^43^. Consistent with our data showing increased JNK phosphorylation in *Mtb*-infected THP-ΔPPM1A macrophages, inhibition of JNK activity by SP600125 abrogated the ability of THP-ΔPPM1A macrophages to undergo apoptosis in response to *Mtb* infection (Fig. 4F), thereby providing further evidence for the involvement of JNK in the PPM1A-mediated block of macrophage apoptosis.

These data demonstrate that modulation of PPM1A levels controls apoptosis of *Mtb*-infected macrophages and suggest that PPM1A could be an attractive drug target to eliminate persistently *Mtb*-infected macrophages.

### Inhibition of PPM1A activity increases JNK activation during *Mtb* infection

Given that genetic manipulations of PPM1A expression showed that it controlled JNK activation as a means to inhibit apoptosis of *Mtb*-infected macrophages, we next sought to test whether PPM1A phosphatase activity was required to inactivate JNK signaling. This would also show whether pharmacological perturbations targeting PPM1A could restore proper JNK activation during *Mtb* infection.

**Figure 5:**
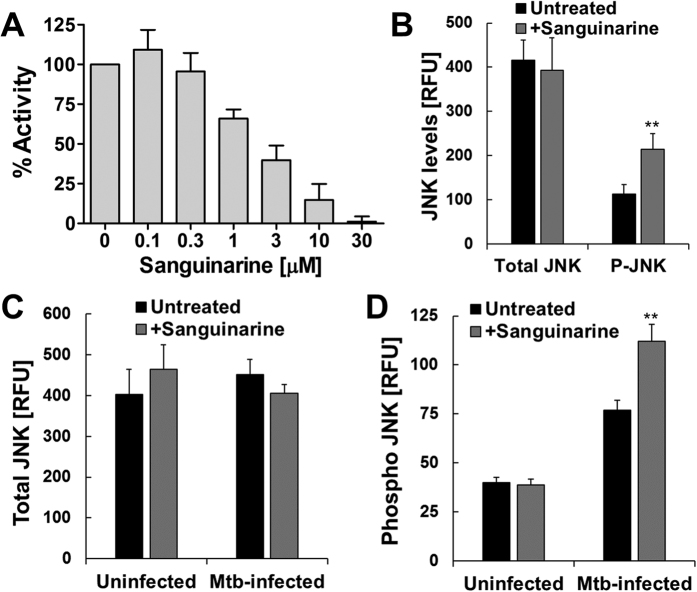
Inhibition of PPM1A activity induces JNK activation. (**A**) Enzymatic activity of PPM1A in the presence of increasing concentrations of sanguinarine was measured by pNPP assay and normalized as 100% in the absence of sanguinarine. (**B**) Primary human monocyte derived macrophages (hMDMs) were treated with 1 μM sanguinarine for 24 h, and then stimulated for 2 h with 100 nM anisomycin. Thereafter, total and phosphorylated JNK levels were measured using the bead-based Milliplex assay. (**C**) Total and (**D**) phosphorylated JNK levels were measured by the Milliplex assay in uninfected and *Mtb*-infected hMDMs (MOI 5; 24 h) that were mock treated or pre-treated with 1 μM sanguinarine for 24 h prior to infection. Data in this figure represent the means ± S.D of three independent experiments. **p < 0.01 relative to untreated macrophages.

Consistent with a previous report that showed the plant product sanguinarine as a potent and selective inhibitor of PPM1A activity^44^, we demonstrated that sanguinarine can block PPM1A phosphatase activity (Fig. 5A). Using sanguinarine as a chemical probe, we treated primary human monocyte derived macrophages (hMDMs) for 24 h, and then induced JNK activation with anisomycin. As we would predict, inhibition of PPM1A activity by sanguinarine increased anisomycin induced phosphorylation of JNK by ~2-fold (Fig. 5B). Importantly, sanguinarine treated hMDMs infected with *Mtb* showed ~45% increase in JNK activation, while levels of total JNK remained similar (Fig. 5C,D). These data show that PPM1A activity is essential for inactivation of JNK during *Mtb* infection and suggests that targeting the PPM1A-JNK signaling axis could restore the proper apoptotic response in *Mtb*-infected macrophages.

### Anisomycin induces selective cell death of *Mtb*-infected primary human macrophages

While we had shown that PPM1A in THP-1 cells and primary human macrophages were similarly upregulated in response to *Mtb*-infection, resulting in a severe impairment of the innate immune response^21^, we would have to verify that this similarity would extend to the ability of PPM1A to control apoptosis of primary macrophages in particular as a function of the identified PPM1A-JNK signaling axis.

Differentiated primary macrophages are highly adherent, which requires aggressive manipulations to de-adhere the cells for downstream single cell analysis. Also, these methods are not conducive to study the kinetic effects of drugs on cell viability. To enable direct kinetic measurements of the effect of chemical research probes on the viability of the *Mtb*-infected primary macrophages, we used Real Time Cell Analysis (RTCA), a method that is frequently used to kinetically determine viability of adherent cells^45^,^46^,^47^. The RTCA system measures changes in impedance between electrodes at the bottom of E-well plates, which is then translated into a Cell Index (CI) measurement, a dimensionless value. An increase in the CI reflects an increase in macrophage adherence (differentiation), whereas a decrease in the CI reflects the loss of macrophage viability as they detach from the bottom of the well (Supplemental Fig. 3). Suspension cells trigger no measurable signal in this system.

To address the question whether interference with the PPM1A-JNK axis would provide a means to selectively kill *Mtb*-infected macrophages, we infected hMDM with *Mtb*, and 24 h post infection, treated the macrophages with or without anisomycin, and then used changes in the CI to kinetically quantify compound effects. Briefly, monocytes were seeded in 96 E-well plates and monitored for GM-CSF induced differentiation. As indicated by the increase in the cell index, primary human monocytes attached to the E-well plate over the first ~140 h (6 days) in response to GM-CSF and differentiated into hMDMs. The hMDMs were left untreated, were exposed to 3 μm inert latex bead particles (phagocytosis control) or infected with *Mtb*. Wells for each condition were then mock treated or treated with anisomycin (75 nM) and macrophage viability (adherence) was followed kinetically by RTCA for the next 24 h to record changes in the CI.

**Figure 6:**
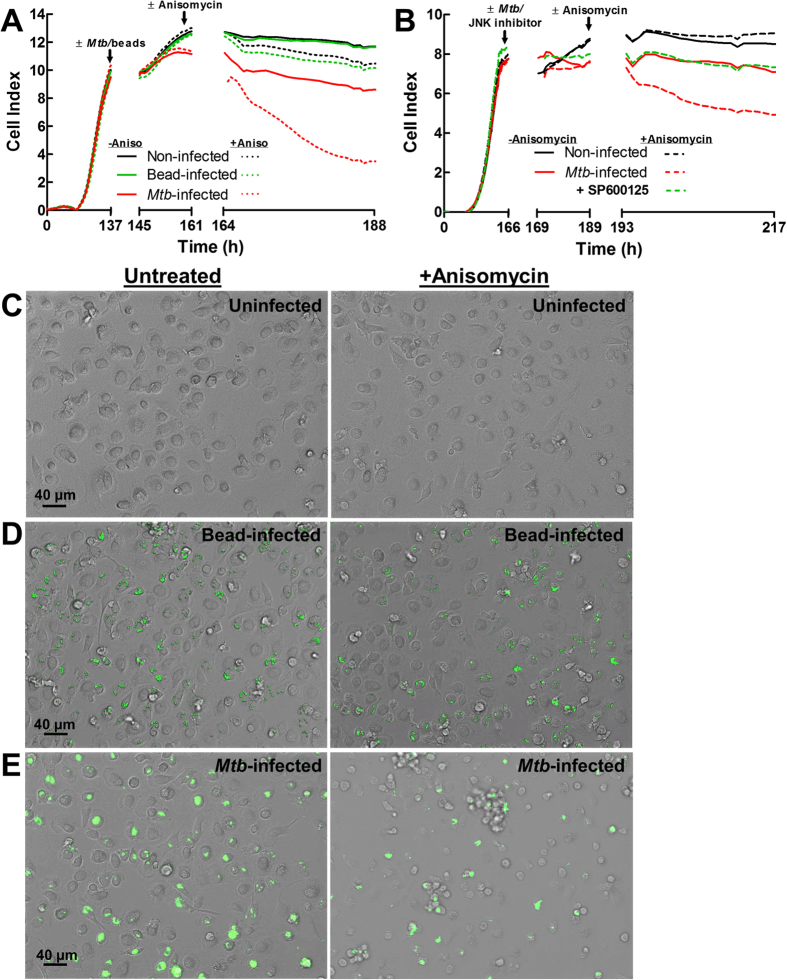
Anisomycin induces selective cell death of *Mtb*-infected primary human macrophages. (**A**) Human monocytes were seeded into E-well plates and GM-CSF (5 ng/ml) differentiation into MDMs was monitored by RTCA to measure cellular adherence as indicated by the Cell Index. Following differentiation (~140 h), MDMs were infected with *Mtb* (MOI 5) or 3 μm latex bead for 24 h, at which point 75 nM anisomycin was added to corresponding wells (~161 h), and changes in the Cell Index was followed for the next 24 h. (**B**) hMDMs were differentiated as in (**A**) and left uninfected or infected with *Mtb* (MOI 5) in the absence or presence of 20 μM SP600125, a specific JNK inhibitor, for 24 h. Thereafter, 75 nM anisomycin was added and changes in the Cell Index was followed for 24 h. CelI Index measurements in (**A,B**) were made every 30 min and represent the average of 3 independent wells. (**C**) Representative bright field images of human MDMs mock treated or treated with 75 nM anisomycin for 24 h. (**D,E**) Representative merged bright field and GFP channel images of (**D**) fluorescent 3 μm latex beads or (**E**) *Mtb*-GFP (MOI 5) infected MDMs following 24 h treatment with or without 75 nM anisomycin.

While the CI remained stable for all control conditions (no anisomycin), and for pseudo-infected MDMs that had phagocytosed the inert latex beads, we observed a selective and rapid decrease in CI in anisomycin treated *Mtb*-infected hMDMs (Fig. 6A). The selective killing of *Mtb*-infected macrophages was indicated by the decrease in CI from 11 to 4, while the CI of untreated *Mtb*-infected macrophages remained above 10 (Fig. 6A). The RTCA experiment demonstrates that *Mtb*-infected primary macrophages were efficiently and selectively killed by the use of anisomycin as a JNK agonist. To unequivocally show that anisomycin induced selective killing of *Mtb*-infected macrophages is not mediated through a p38 dependent mechanism and that JNK activation is absolutely required in this process, we used a specific JNK inhibitor, SP600125^43^ to probe the system. Using RTCA as described above, we show that the presence of SP600125 completely blocked the ability of anisomycin to induce selective apoptotic killing of *Mtb*-infected human macrophages (Fig. 6B). This result demonstrates that anisomycin induced selective cell death can be reversed by directly inhibiting JNK.

The RTCA data were confirmed by fluorescence microscopy analysis in an independent experiment. On day 6 post GM-CSF differentiation, macrophage differentiation was indicated by a strongly adherent, flattened, pancake-like morphology indicative of M1 polarization^48^, which was not altered by the addition of anisomycin (Fig. 6C). Similarly, no influence of anisomycin on the viability or phenotype of hMDMs loaded with 3 μm inert latex bead particles was observed (Fig. 6D). However, anisomycin treatment of *Mtb*-infected macrophages resulted in extensive cell death compared to *Mtb*-infected macrophages in the absence of anisomycin (Fig. 6E). While untreated *Mtb*-infected macrophages maintained the flattened, pancake-like morphology, anisomycin treated *Mtb*-infected macrophages exhibited features of cell shrinkage, detachment, and formation of apoptotic bodies with some extracellular bacteria, typical features of apoptotic cells (Fig. 6E).

Taken together, these data suggest that selective killing of *Mtb*-infected macrophages can be achieved by pharmacological perturbation and that targeting the PPM1A-JNK signaling network is a promising therapeutic strategy to induce selective elimination of persistently *Mtb*-infected macrophages.

### Selective killing of *Mtb*-infected macrophages enhances antimicrobial chemotherapy to kill *Mtb*

Apoptosis of infected macrophages stimulates the antibacterial response^5^ and plays a significant role in promoting adaptive immunity^3^. Alternatively, given reports that several key TB drugs such as rifampicin^26^,^27^, streptomycin, and fluoroquinolones have decreased efficacy against intracellular *Mtb* likely due to penetration properties^29^, induction of apoptosis may also improve the ability of existing antimicrobial chemotherapy against *Mtb*.

To test this hypothesis, *Mtb*-infected THP-1 macrophages were treated with anisomycin to induce apoptosis-mediated bacterial release, and subsequently treated with rifampicin at a concentration below the minimal inhibitory concentration (MIC) necessary to kill intracellular *Mtb*.

**Figure 7:**
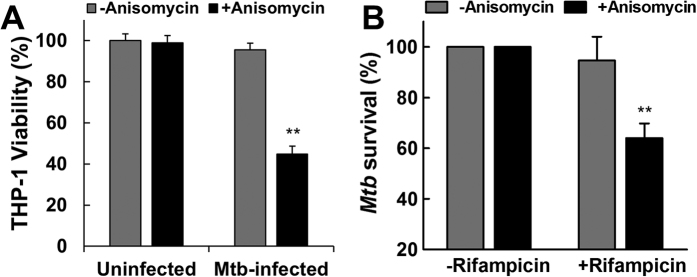
Selective killing of *Mtb*-infected macrophages by anisomycin enhances killing of *Mtb* by rifampicin. (**A**) THP-1 macrophages were uninfected or infected with *Mtb* (MOI 5) for 24 h, and subsequently mock treated or treated with 100 nM anisomycin for 72 h. THP-1 macrophage viability was then measured using CellTiter-Glo luminescent assay and normalized to uninfected, untreated cells as 100% viability. (**B**) *Mtb*-infected THP-1 macrophages (MOI 5) were treated with or without 100 nM anisomycin for 3 days. Then cells were treated with or without 0.1μg/ml rifampicin for 1 day. The amount of viable *Mtb* in each condition was quantified using the luciferase activity assay. Using relative luminescence units, data were normalized to control conditions (without rifampicin) as 100% *Mtb* survival. Results in this figure represent the means ± S.D. of three independent experiments. **p < 0.01 relative to untreated macrophages.

Consistent with our data using primary human macrophages, we show that anisomycin treatment induces selective killing of *Mtb*-infected THP-1 macrophages (Fig. 7A). Subsequently, to measure *Mtb* viability, we used a well-characterized luciferase reporter system in *Mtb*^49^,^50^, which has been shown to correlate well with the standard colony unit formation plating method^51^. Our experiments show that while anisomycin or rifampicin at the used concentration had little to no effect on the killing of intracellular *Mtb*, combination treatment decreased the survival of *Mtb* by ~40% as measured by luciferase assays (Fig. 7B). This experiment suggests that selective induction of *Mtb*-infected macrophage apoptosis improves standard antimicrobial chemotherapy and should provide an attractive treatment strategy that has the potential to shorten the currently lengthy treatment period.

To explore the possible effect of such a “release and kill” strategy on TB treatment kinetics, we used a simple mathematical model that assumes a biphasic decline of the bacterial load in patients following onset of chemotherapy (e.g. rifampicin treatment) (Supplemental Fig. 4). Based on a series of data published by other groups^52^,^53^,^54^, we assumed that during phase I (initial kill), therapy would eliminate extracellular *Mtb* and intracellular *Mtb* in macrophages located in tissues where sufficiently high drug concentrations can be easily achieved. During this phase, a reduction of the bacterial burden by ~70% would be achieved within a month of standard treatment. Phase II (slow kill) would be characterized by a much slower decline of the residual bacterial burden, which under standard treatment would be completely eliminated after ~6 months (24 weeks) of treatment (Supplemental Fig. 4; black curve). This slow decline would be representative of the effect of drug treatment against intracellular *Mtb*, possibly in a persistent and therefore more therapy resistant form and in tissue locations that are poorly penetrated by drugs. The model provided us with the ability to predict various theoretical treatment scenarios, in which *Mtb*-release drugs that trigger selective apoptosis of *Mtb*-infected macrophages would be added as adjunctive therapy to the ongoing standard *Mtb* treatment regimen.

In Supplemental Fig. 4, several scenarios are visualized. In a scenario where release drugs are not added to the regimen during phase I or their addition would not increase phase I treatment efficacy, this model would predict that a weekly application schedule of *Mtb*-release drugs that would result in a 10%, 20%, or 40% reduction in the bacterial burden (based on what can be achieved in Fig. 7B) would lead to a reduction in treatment times from 24 weeks to 15, 12, or 9 weeks, respectively. If *Mtb-*release drugs were to improve phase I treatment efficacy by 10% or 20%, and subsequently reduce the *Mtb* burden by even only 10%/week during phase II, treatment times would be reduced to 13 or 9 weeks, respectively. While these are only possible predictions based on simple, but realistic assumptions, these calculations suggest that a release and kill strategy-based adjunctive therapy to the standard TB treatment regimen could lead to a major reduction in treatment time.

## Discussion

*Mtb* is a prototypical intracellular bacterial pathogen that replicates efficiently in the phagosome of macrophages^55^. We recently demonstrated that to enable this process, *Mtb* alters the kinase and phosphatase signaling networks within the infected macrophage resulting in an environment conducive to the establishment of a persistent infection. We found that *Mtb*-induced upregulation of PPM1A controls several aspects of the macrophage innate antibacterial response^21^.

In the current study, we demonstrate that PPM1A is an apoptosis checkpoint that controls both the intrinsic and extrinsic apoptosis pathways (Fig. 1), a critical innate immune response with major implications for intracellular pathogens. Kinome analysis enabled the identification of the JNK/AP-1 signaling pathway as a downstream effector through which PPM1A exerts its control on the apoptotic pathway in macrophages (Supplemental Fig. 1 and Table 1), which was confirmed by a series of experiments using PPM1A overexpressing and knockdown cells (Figs 3 and 4). These experiments revealed that the ability to control PPM1A expression provided *Mtb* with a means to hijack macrophages by suppressing host cell apoptosis. Suppression of apoptosis, which is otherwise an innate defense mechanism against intracellular pathogens, provides *Mtb* with a replicative niche within which it can persist for long periods. Indeed, apoptosis of infected macrophages has been shown to facilitate intracellular bacterial killing, priming of cell mediated immunity, and limits unnecessary tissue inflammation^2^,^3^,^5^,^6^,^7^. Suppression of apoptosis is also essential for eventual *Mtb* dissemination, which is achieved by *Mtb*-induced host cell necrosis. PPM1A upregulation thus seems to be a key factor for *Mtb* to establish a persistent infection in macrophages, by hijacking the innate immune response of its host cells, and disabling the natural ability of macrophages to undergo apoptosis in response to pathogen infection.

Our finding that a host phosphatase (PPM1A) controls both the extrinsic and intrinsic apoptotic pathways, and more importantly inhibits apoptosis of *Mtb*-infected macrophages corroborates with research on other members of the PPM (monomeric, metal-dependent) family of serine/threonine phosphatases^56^, which have well described functions in the regulation of cell death^23^,^30^,^57^. In addition, it was recently demonstrated that the *Mtb* phosphatase PtpA could dephosphorylate host Glycogen Synthase Kinase-3 (GSK3α), thereby inhibiting apoptosis of *Mtb*-infected macrophages^38^. Consistent with this, kinome analysis from persistently *Mtb*-infected THP-1 cells^21^ and that of PPM1A overexpressing cells (Supplemental Fig. 1) both show dysregulation of GSK3. There is also evidence showing that *Mtb* PtpA exploits host ubiquitin to inactivate JNK and p38, thereby suppressing innate immunity^58^. Our finding that PPM1A controls the innate immune response^21^ and apoptosis during *Mtb* infection may suggest the existence of redundant mechanisms (via host and bacterial phosphatases) used by *Mtb* to ensure survival of the host macrophage. Such functional redundancy would also explain why knockdown of PPM1A in our experiments induced apoptosis in a large proportion of, but not all *Mtb*-infected macrophages.

As the PPM1A-JNK signaling axis presented itself as an attractive target to restore the ability of *Mtb*-infected macrophages to undergo apoptosis, we also address the important question whether this signaling network could be targeted to selectively kill *Mtb*-infected macrophages. Such a therapeutic intervention would (i) disrupt the intracellular replication cycle of *Mtb*, (ii) boost the adaptive immune response to *Mtb*^8^, and (iii) increase the susceptibility of *Mtb* to existing drug treatments through induction of macrophage apoptosis given that common anti-tuberculosis drugs (e.g. rifampicin) have reduced efficacy against intracellular bacteria^26^,^27^.

Given that we had identified JNK as a key downstream signaling effector of PPM1A, it was logical to target JNK to deliver proof of concept that selective apoptosis induction of *Mtb-*infected macrophages can be achieved. Indeed, anisomycin, a reported JNK phosphorylation agonist could counteract the PPM1A upregulation enforced suppression of JNK phosphorylation following *Mtb*-infection, and restored the ability of macrophages to undergo apoptosis in response to *Mtb* infection (Fig. 6).

Drugs that could selectively induce apoptosis of *Mtb*-infected macrophages are highly desirable as this would restore a key innate immune response that is actively blocked by *Mtb* infection, which will have therapeutic benefits through the generation of an improved adaptive immune response and the ability to potentiate vaccines. Beyond this, drug-induced selective killing of *Mtb*-infected macrophages could likely increase the efficacy of existing anti-*Mtb* drugs as they gain better access to the bacteria. This approach would be particularly useful as adjunctive TB therapy since the commonly used anti-*Mtb* drugs rifampicin^26^,^27^, streptomycin, and fluoroquinolones having decreased efficacy against intracellular *Mtb* likely due to penetration properties^29^. Indeed, in a process we term “release and kill”, our results show that anisomycin induced killing of *Mtb*-infected macrophages removes the protective niche of the bacteria and increases the efficacy of rifampicin to kill *Mtb* at concentrations that are normally inactive against intracellular *Mtb*.

To examine the possible impact of the addition of *Mtb*-release drugs to standard TB treatment regimens, a simple mathematic model that allowed for some basic predictions suggested that a shortening of the treatment course by 35–60% seemed rather realistic. As clinical anti-tuberculosis drug treatment, particularly in resource poor settings, is primarily hampered by long treatment periods lasting over 6 months^59^ leading to patient non-adherence, any significant shortening of the treatment period would be highly desirable.

## Methods

### Cell culture, reagents, and antibodies

THP-1 monocytes (ATCC TIB-202) and primary monocytes were maintained in RPMI 1640 medium supplemented with 2 mM L-glutamine and 10% heat-inactivated fetal bovine serum (FBS) at 37 °C in a humidified atmosphere of 5% CO_2_. THP-PPM1A cells were generated previously^21^. Human PBMCs were isolated from buffy coats by the Ficoll-Paque density centrifugation method. Monocytes were enriched by positive selection using anti-CD14 mAb-coated microbeads from Miltenyi Biotec (San Diego, CA) according to manufacturer’s protocol. Monocytes were then differentiated with 5 ng/ml GM-CSF (R&D Systems, Minneapolis, MN) for 5–7 days to obtain monocyte derived macrophages (MDM). Fetal bovine serum was obtained from Life Technologies (Grand Island, NY). Phorbol ester 13-phorbol-12-myristate acetate (PMA), puromycin, etoposide, kanamycin and anisomycin were purchased from Sigma (St. Louis, MO). Sanguinarine and SP600125 were purchased from Fisher Scientific (Pittsburgh, PA). CellTiter-Glo luminescent cell viability assay kit was purchased from Promega (Madison, WI). Polyclonal PPM1A antibodies were purchased from Thermo Scientific (Rockford, IL). Monoclonal mouse antibodies to GAPDH and α-tubulin were purchased from Santa Cruz (Dallas, Texas) and Cell Signaling (Danvers, MA), respectively.

### Bacteria and plasmids

The *M. tuberculosis* H37Rv derived auxotroph strain mc^2^6206 was grown in Middlebrook 7H9 medium (Difco) supplemented with 0.2% glycerol, 0.02% Tyloxapol and 10% OADC (Remel) or on Middlebrook 7H10 plates supplemented with 0.5% glycerol and 10% OADC (Remel). Growth media of the auxotrophic *M. tuberculosis* strain was supplemented with 24 μg/ml pantothenate and 50 μg/ml L-leucine^60^. Hygromycin B was purchased from Calbiochem. *gfp* expressing *M. tuberculosis* mc^2^6206 was generated previously^21^. *M. tuberculosis* mc^2^6206 expressing luciferase was generated by transformation of the pJAK.1-luciferase plasmid^49^ (generous gift from Dr. Hmama). *Escherichia coli* strain DH5α was used for plasmid propagation, and was routinely grown in Luria-Bertani broth at 37 °C. Lentiviral vectors expressing shRNA targeting human PPM1A were generated by Genecopoeia (Rockville, MD) using the sequences: shRNA #1: 5′-GTACCTGGAATGCAGAGTA and shRNA #2: 5′- GTCGACACCTGTTTGTATA. THP-ΔPPM1A cells generated using shRNA #2 showed the best knockdown and was used for all subsequent experiments.

### Generation of THP-ΔPPM1A cells

HEK 293T cells seeded at 50% confluency were transfected with lentiviral plasmids expressing shRNA targeting human PPM1A or scrambled control using FuGENE (Promega, Madison, WI). Culture supernatants were harvested after 48 h and 72 h, aliquoted, and stored at −80 °C. The supernatants containing lentiviral particles were used to transduce THP-1 cells. Cells were selected by puromycin and analyzed by Western blot to verify knockdown of PPM1A protein levels.

### Bacterial infection

*M. tuberculosis* mc^2^6206 growing in log-phase was quantified by optical density measurement at 600 nm using the conversion of 3 × 10^8^ (*Mtb*) bacteria per ml for OD 1.0. The amount of bacteria required for various MOIs were washed and resuspended in RPMI 1640 cell culture media without antibiotics. For infection of THP-1 monocytes, the prepared bacteria were added to the THP-1 cells and resuspended together to ensure even exposure of the monocytes to bacteria. For infection of differentiated THP-1 macrophages and primary human MDMs, bacteria were added to the cells and incubated at 37 °C for 4 h. Extracellular, non-phagocytosed bacteria were removed by 3 washes and infection was continued 37 °C for the desired time.

### Survival of *Mtb* during macrophage infection

Entire wells of *Mtb*-infected macrophages were harvested by collecting the cells in the supernatant and cells that were still attached to the well bottoms 3 days post infection. These fractions were combined and macrophages were lysed in Glo Lysis Buffer (Promega) to measure the amount of viable *Mtb* in each well. Luciferase activity, proportional to bacterial load, was determined by using the BrightGlo Luciferase Assay System (Promega) according to the manufacturer’s protocol. Resultant luminescence was measured with the Cytation 3 Multi-Mode Reader (BioTek, Winooski, VT) using 96-well solid white plates (Corning, Corning, NY) and an integration time of 1 s per well.

### Apoptosis assays

Cells were stained with Annexin V conjugated to AlexaFluor 488 or APC (Life Technologies) according to manufacturer’s protocol. FAM FLICA™ (green) or FLICA™ 660 (far red) caspase-3/7 staining kits were used to stain for active caspase-3/7 in cells, and were used according to manufacturer’s protocol (ImmunoChemistry Technologies, Bloomington, MN). Apo-ONE homogenous caspase-3/7 detection kit was purchased from Promega, and used according to manufacturer’s protocol.

### Flow cytometry

Cells following Annexin V or FLICA staining were analyzed by flow cytometric analysis (FCM). FCM analysis was performed on a Guava EasyCyte (Guava Technologies Inc., Billerica, MA). Data analysis was performed using Guava Express software (Guava Technologies Inc.) or FlowJo V10 software (Ashland, OR).

### Milliplex assays

Cells following various treatments or *Mtb* infections were lysed according to Milliplex kit protocol (Millipore, Billerica, MA). Total and phosphorylated JNK (T183/Y185) or p38 (T180/Y182) protein levels were measured using separate 2-plex Milliplex kits. Experiments were performed according to manufacturer’s protocol and read-out was performed using a Bio-Plex 200 instrument. Data analysis was performed using Bio-Plex manager 6.0 software (Bio-Rad, Hercules, CA).

### PPM1A activity assay

The enzymatic activity of PPM1A was measured using the pNPP phosphatase assay kit (BioAssay Systems, Hayward, CA). In brief, 10 μg/ml recombinant PPM1A (Abnova, Walnut, CA) was prepared in reaction buffer (25 mM HEPES, 100 mM NaCl, 2 mM MnCl_2_, pH 7.2) in the absence or presence of 0–30 μM sanguinarine for 15 min at room temperature. The resulting reaction was mixed with pNPP substrate mix according to manufacturer’s protocol and incubated at 37 °C for 15 min. The reaction was then stopped using the kit provided stop solution and absorbance was measured in a plate reader at 405 nm.

### Real-time cell analyzer assay (RTCA)

Macrophage adhesion was measured in specialized 96-well plates (E-plate 96) with the xCELLigence Real-time Cell Analyzer (RTCA) SP apparatus (ACEA Biosciences, San Diego, CA). Data was quantified by measuring impedance changes between the sensing electrodes located in the well-bottom, which changes as a function of the adhesion of cells to the surface of the plate. A dimensionless value, the Cell Index (CI), is representative of these impedance changes. Using this system, macrophage adhesion and therefore viability was monitored in real-time. Plates were removed at various time points for the addition of anisomycin or *Mtb* for infection. After each manipulation, plates were placed back in the RTCA apparatus for kinetic monitoring. The CI at every time point represents the mean of three independent measurements. A schematic description of this system is shown in Supplemental Fig. 3.

### Western blot

Cells were harvested by centrifugation, washed once with PBS, and lysed in RIPA buffer (Cell Signaling) according to the manufacturer’s instructions. Protein concentration of the lysates was determined by the bicinchoninic acid (BCA) method according to the manufacturer’s recommendations (Thermo Scientific). About 10 to 20 μg of protein per sample was separated on 10% Mini-Protean TGX gels (Bio-Rad) and subsequently transferred to a polyvinylidene difluoride (PVDF) membrane using an iBlot gel transfer system (Life Technologies). Western blot analysis was performed according to standard protocols. Total PPM1A, α-tubulin, or GAPDH proteins were detected with specific antibodies (see antibody section). A horseradish peroxidase-conjugated goat anti-rabbit or goat anti-mouse polyclonal antibody (Santa Cruz) was used as the secondary antibody. The blot was developed using the Western Lightning Ultra chemiluminescent substrate from Perkin Elmer, Inc., and detected in an EpiChemi3 darkroom (UVP BioImaging Systems).

### Kinex™ Antibody Microarray-based analysis

Kinex™ antibody array analysis was performed as before^21^. Briefly, 50 μg of lysate from each sample were covalently labeled with a proprietary fluorescent dye according to the manufacturer’s instructions (Kinexus, Canada). After blocking non-specific binding sites on the array, an incubation chamber was mounted onto the microarray to permit the loading of one control (THP-1) and one experiment (THP-PPM1A) sample side-by-side on the same chip. Following sample incubation, unbound proteins were washed away. KAM-850 chips are spotted in duplicates with over 850 antibodies, including 517 pan-specific antibodies and 337 phosphosite-specific antibodies. By this means, the microarrays provide information about the expression and phosphorylation levels of these target proteins.

Each array produces a pair of 16-bit images captured with a Perkin-Elmer ScanArray Reader laser array scanner (Waltham, MA). Signal quantification was performed with ImaGene 8.0 from BioDiscovery (El Segundo, CA) with predetermined settings for spot segmentation and background correction. The background-corrected raw intensity data are logarithmically transformed with base 2. Z-scores are calculated by subtracting the overall average intensity of all spots within a sample from the raw intensity for each spot, and dividing it by the standard deviations (SD) of all of the measured intensities within each sample^61^. Z-ratios are further calculated by taking the difference between the averages of the observed protein Z-scores and dividing by the SD of all of the differences for that particular comparison. Calculated Z-ratios have the advantage that they can be used in multiple comparisons without further reference to the individual conditional standard deviations by which they were derived.

### Statistical analysis

Data are expressed as the mean ± the standard deviation of three independent experiments. Statistical analysis was performed using the Student *t* test. Values of *p* < 0.05 were considered to be significant.

## Additional Information

**How to cite this article**: Schaaf, K. *et al*. *Mycobacterium tuberculosis* exploits the PPM1A signaling pathway to block host macrophage apoptosis. *Sci. Rep.* **7**, 42101; doi: 10.1038/srep42101 (2017).

**Publisher's note:** Springer Nature remains neutral with regard to jurisdictional claims in published maps and institutional affiliations.

## Supplementary Material

Supplementary Figures 1–4

## Figures and Tables

**Table 1:** Differentially expressed proteins in THP-PPM1A cells involved in the regulation of apoptotic processes.

Target Protein Name	Phospho Site (Human)	Globally Normalized -THP-1	Globally Normalized -THP-PPM1A	Z-ratio (THP-PPM1A/THP-1)	Uniprot Link
JNK1/2/3	Pan-specific	8182	1720	**−3.80**	P45983
MEK1 (MAP2K1)	Pan-specific	1911	521	**−3.39**	Q02750
ErbB2 (HER2)	Pan-specific	7069	1809	**−3.35**	P04626
KHS (MAP4K5)	Pan-specific	2960	923	**−3.01**	Q9Y4K4
PKR1	Pan-specific	2953	1097	**−2.59**	P19525
Lck	Pan-specific	861	353	**−2.54**	P06239
PP2A/Ca	Pan-specific	599	252	**−2.53**	P67775
Hsp105	Pan-specific	9909	3539	**−2.52**	Q92598
Hpk1 (MAP4K1)	Pan-specific	13234	4744	**−2.47**	Q92918
Hsp27	S82	4186	1617	**−2.45**	P04792
Mcl1	Pan-specific	8514	3241	**−2.39**	Q07820
Plk2	Pan-specific	5303	2089	**−2.37**	Q9NYY3
PP2A B’ (B56)	Pan-specific	12073	4679	**−2.29**	Q15172
LAR	Pan-specific	1450	648	**−2.26**	P10586
Fos	Pan-specific	15992	6482	**−2.15**	P01100
Jun	Pan-specific	981	481	**−2.09**	P05412
COX2	Pan-specific	783	399	**−2.04**	P35354
JNK1/2/3	Pan-specific	731	383	**−1.98**	P45983
Hsc70	Pan-specific	7101	3320	**−1.93**	P11142
PKCm (PKD)	Pan-specific	2313	1167	**−1.91**	Q15139
PKBb (Akt2)	Pan-specific	4790	2369	**−1.85**	P31751
CDK1 (CDC2)	Pan-specific	6759	3391	**−1.77**	P06493
PKBa (Akt1)	Pan-specific	13995	6781	**−1.75**	P31749
HO1	Pan-specific	15873	7684	**−1.73**	P09601
Hsp27	S78	14306	7090	**−1.69**	P04792
eIF2a	S52	2743	1506	**−1.68**	P05198
Hsp90a/b	Pan-specific	11594	5938	**−1.64**	P07900
Lck	Pan-specific	6160	3315	**−1.61**	P06239
Catenin b1	Pan-specific	18725	9440	**−1.61**	P35222
IRAK1	Pan-specific	8278	4572	**−1.51**	P51617
CDK1 (CDC2)	Pan-specific	7669	4303	**−1.48**	P06493
FRS2	Y348	5084	2931	**−1.48**	Q8WU20
PDK1	Pan-specific	578	382	**−1.46**	O15530
PKCh	Pan-specific	6323	3633	**−1.46**	P24723
PKR1	T446	2498	1535	**−1.43**	P19525
PKCm (PKD)	S738 + S742	765	511	**−1.40**	Q15139
Hsc70	Pan-specific	23652	12908	**−1.39**	P11142
CASP1	Pan-specific	6292	3775	**−1.35**	P29466
Hsp60	Pan-specific	27986	15480	**−1.34**	P10809
PKBb (Akt2)	Pan-specific	5431	3325	**−1.33**	P31751
Lyn	Pan-specific	9390	5674	**−1.28**	P07948
CDK1 (CDC2)	Pan-specific	11030	6640	**−1.27**	P06493
Abl	Pan-specific	1510	1048	**−1.21**	P00519
Hsp60	Pan-specific	10753	6639	**−1.21**	P10809
STAT1a	Pan-specific	421	886	**1.23**	P42224
FAK	Y576/Y577	2946	5519	**1.23**	Q05397
Hsp27	S15	480	1020	**1.27**	P04792
MEK1 (MAP2K1)	T292	1028	2108	**1.29**	Q02750
Jun	S73	5622	10590	**1.33**	P05412
Hsp90a/b	Pan-specific	1144	2394	**1.36**	P07900
GSK3b	Pan-specific	350	787	**1.36**	P49841
IRAK2	Pan-specific	3275	6557	**1.40**	O43187
CDK5	Pan-specific	674	1509	**1.44**	Q00535
Grp94	Pan-specific	407	949	**1.46**	P14625
ErbB2 (HER2)	Pan-specific	226	548	**1.48**	P04626
IGF1R	Pan-specific	244	592	**1.48**	P08069
GSK3a	Y279	624	1430	**1.48**	P49840
HDAC4	Pan-specific	719	1649	**1.51**	P56524
GSK3b	Pan-specific	453	1074	**1.52**	P49841
NR1	S896	2774	5896	**1.52**	Q05586
CAMK2d	Pan-specific	398	951	**1.52**	Q13557
MEK2 (MAP2K2)	Pan-specific	412	989	**1.53**	P36507
NFkappaB p65	S529	871	2018	**1.56**	Q04206
PKCd	S645	834	1957	**1.58**	Q05655
GSK3a	Pan-specific	420	1044	**1.62**	P49840
Hsp90a/b	Pan-specific	591	1445	**1.63**	P07900
TAO3 (JIK)	Pan-specific	488	1228	**1.67**	Q9H2K8
Jun	S243	697	1719	**1.68**	P05412
GroEL	Pan-specific	4427	9914	**1.71**	P10809
CREB1	S133	1042	2583	**1.74**	P16220
Nek4	Pan-specific	277	753	**1.77**	P51957
NFkappaB p65	S536	2603	6280	**1.81**	Q04206
GNB2L1	Pan-specific	600	1616	**1.86**	P63244
PKBa (Akt1)	S473	1014	2770	**1.97**	P31749
CDK5	Pan-specific	1175	3245	**2.02**	Q00535
FAK	S732	1081	3471	**2.36**	Q05397
FAK	S843	933	3371	**2.62**	Q05397
NFkappaB p65	S276	590	2483	**2.91**	Q04206
