# Supplementary material for: Mycobacterium tuberculosis exploits the PPM1A signaling pathway to block host macrophage apoptosis
Source: Sci Rep. 2017 Feb 8;7:42101. doi: 10.1038/srep42101 (PMC5296758; doi:10.1038/srep42101)
Supplement: Supplementary Figures 1–4 [file srep42101-s1.doc]

**SUPPLEMENTARY INFORMATION**

***Mycobacterium tuberculosis* exploits the PPM1A signaling pathway to block host macrophage apoptosis**

Kaitlyn Schaaf, Samuel R Smith, Alexandra Duverger, Frederic Wagner, Frank Wolschendorf, Andrew O. Westfall, Olaf Kutsch*, and Jim Sun*

Department of Medicine, University of Alabama at Birmingham, Birmingham, Alabama, USA

Correspondence to Jim Sun, email: jsun14[at]uab.edu or Olaf Kutsch, email: olafkutsch[at]uabmc.edu.

**Keywords:** Apoptosis, PPM1A, macrophages, *Mycobacterium tuberculosis*, cell death

**SUPPLEMENTARY FIGURES**

**
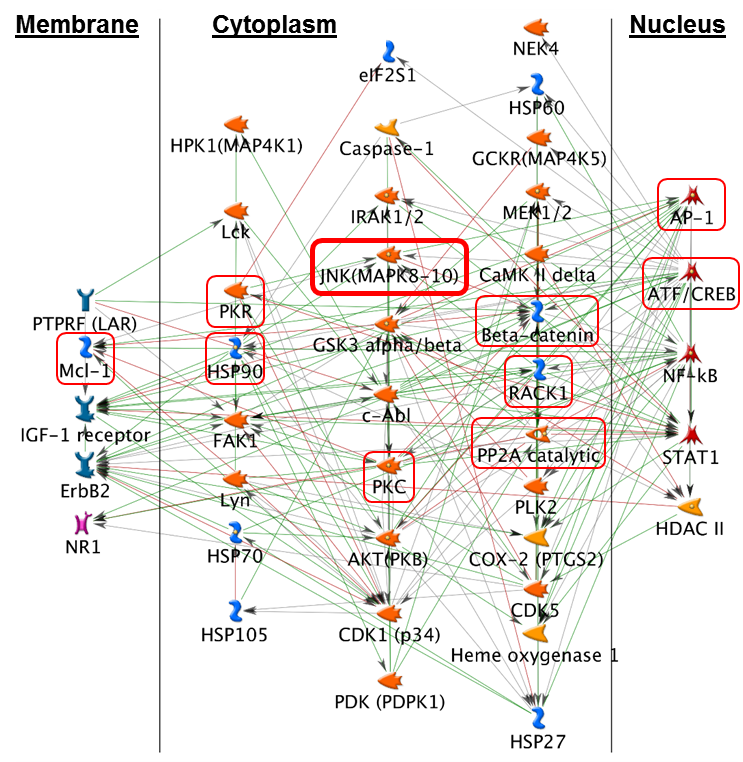
**

**Supplementary Figure 1. Protein-protein interaction network (PIN) of signals identified in kinome analysis of THP-PPM1A cells.** A direct PIN analysis was performed using Metacore with proteins identified from kinome analysis of THP-PPM1A cells as seed nodes. JNK-AP-1 was identified as a highly connected node (red boxes) that could potentially function downstream of PPM1A signaling. Green and red arrows indicate activation or inhibition effects, respectively.


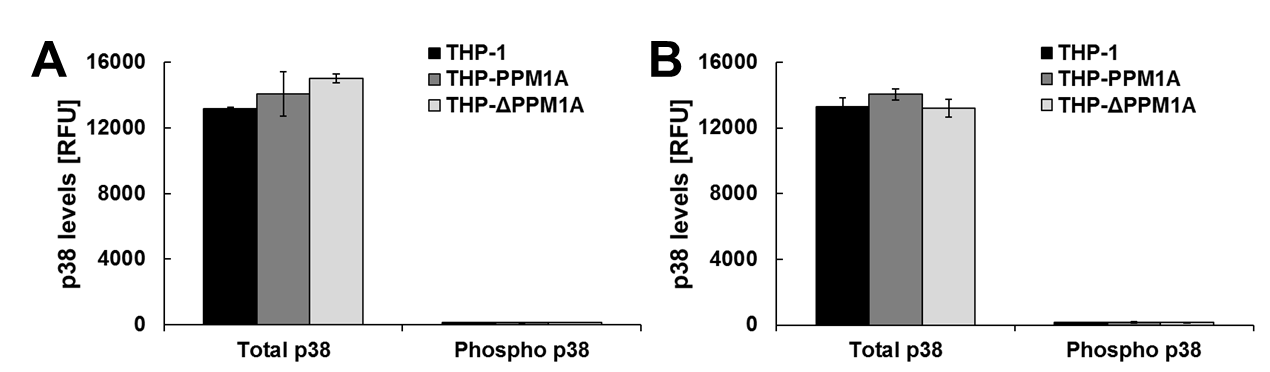


**Supplementary Figure 2. Mtb infection does not alter p38 MAPK protein levels or phosphorylation.** (**A**) Resting THP-1, THP-PPM1A, and THP-ΔPPM1A cells were lysed and the amount of total and activated p38 (phosphorylated; T180/Y182) were measured using a 2-plex Milliplex assay kit. RFU indicates relative levels of total or phosphorylated JNK in these cells at baseline. (**B**) THP-1, THP-PPM1A, and THP-ΔPPM1A cells were infected with *Mtb* at an MOI of 20 for 48 h. Then, cells were lysed and the amount of total and activated p38 were measured using the Milliplex assay. Data in this figure represent the means ± S.D. of three independent experiments.

**
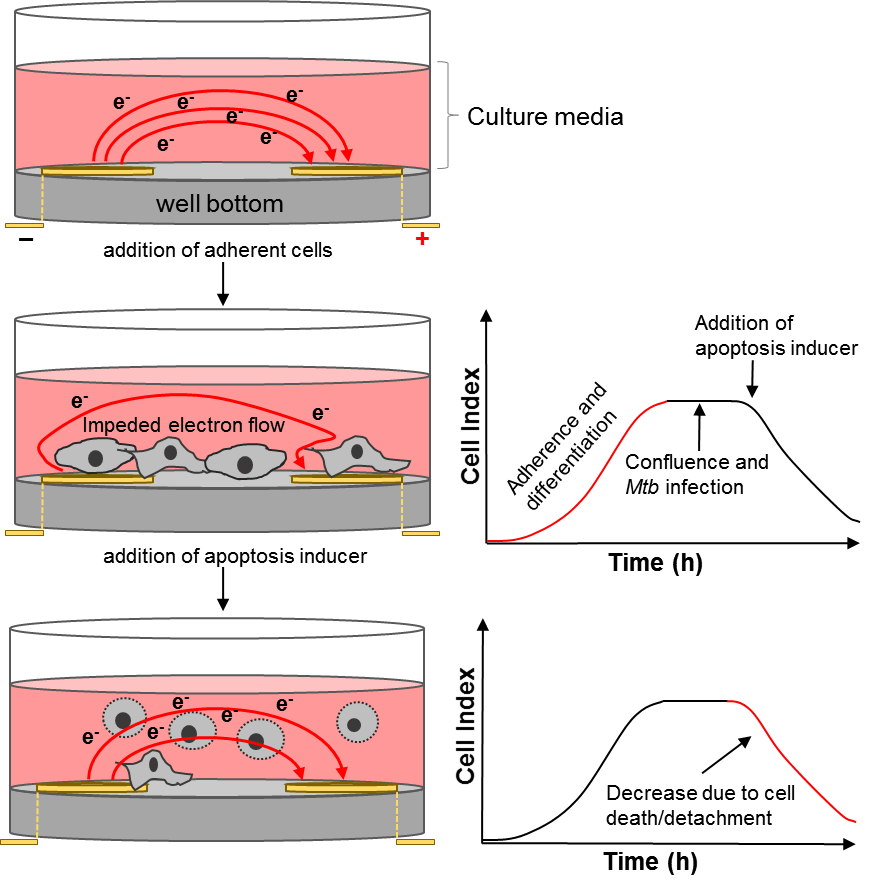
**

**Supplementary Figure 3. Schematic description of using Real-time cell analysis (RTCA) to measure macrophage cell death.** The RTCA system measures changes in impedance between electrodes at the bottom of specialized 96-plates (E-well plates), which is then translated into a Cell Index (CI) measurement, a dimensionless value. As cells adhere to the well bottoms, the current flow between the electrodes is impeded, resulting in an increase in the CI, which reflects macrophage differentiation (adherence). Conversely, upon loss of macrophage viability (addition of apoptosis inducer) there is a corresponding decrease in CI, which reflects the detachment of macrophages from the well bottoms. This system only measures true adherence as suspension cells that settle to the bottom of wells trigger no measurable signal.

**
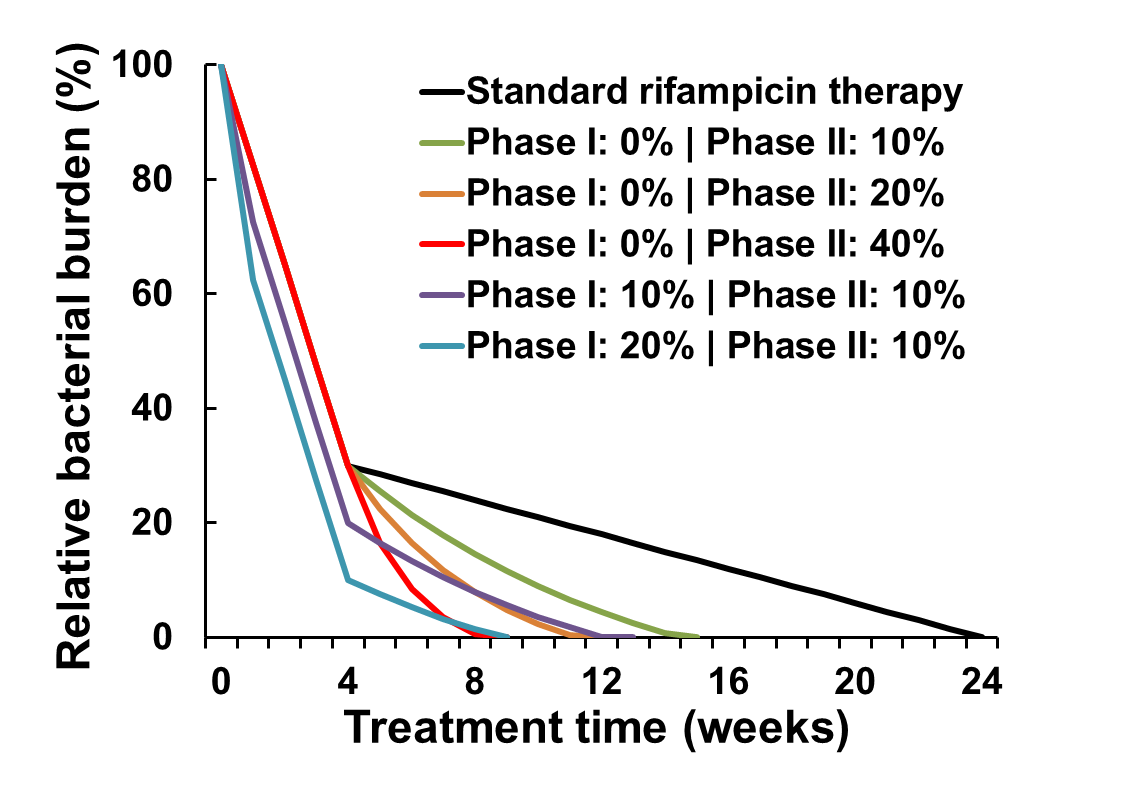
**

**Supplementary Figure 4**. **A mathematic model to predict the potential impact of a release and kill strategy as adjunctive therapy to standard rifampicin treatment against *Mtb* infection.**  A biphasic decline in *Mtb* burden is assumed over the course of 24 weeks of standard rifampicin therapy where an initial rapid killing phase (phase I) results in a 70% decline in *Mtb* burden after 4 weeks, followed by a slow killing phase (phase II) from weeks 5 to 24, when the residual bacterial burden would be completely eliminated (black curve). Different scenarios following weekly addition of *Mtb*-release drugs are visualized assuming no effect in phase I (0%) combined with 10%, 20%, or 40% increased killing of *Mtb* in phase II (green, orange, and red lines). Alternatively, a 10 or 20% increase in Mtb killing during phase I combined with a 10% increase in *Mtb* killing in phase II are also shown (purple and blue lines).
